# Extra Limites
*The *extra limites* department was originally opened for the purpose of giving authors an opportunity of explaining misapprehensions, or of defending themselves against, what they might conceive to be, unjust criticism in this, as well as other Reviews. As the public must not be charged with these defences, authors are hereby informed, that the expence of insertion is *nine shillings per page*, the exact prime cost of paper and printing. All the control exerted, is over language that might be deemed derogatory to science, or beneath the dignity of the profession.


**Published:** 1820-12-01

**Authors:** James Johnson


### EXTRA LIMITES.*

### DR. JOHNSON'S REPLY TO CERTAIN LIBERAL REVIEWERS.

" Sed vident ingratos, intabescuntque videndo,

" Successus hominum ——————————"

When a man is violently assailed by those who are his competitors for public favour, and covered with all kinds of abuse and obloquy, it is a pretty significant symptom of his prosperity, and of the flourishing state of his undertakings, whatever they may happen to be. I could not, therefore, be very much dismayed, in beholding a storm of the lowest ribaldry poured upon me by Drs. Hutchinson and Reece, in the Medical and Physical Journal, and in the Gazette of Health. That I have escaped the humiliation of being praised in the Gazette of Health, must afford satisfaction to all those who wish me well—and that I have not much to dread from the vituperation of the Medical and Physical Journal, will be readily granted by those who read that work, and who remember that only two short years ago, I was there stated "to possess a strong claim upon the approbation and esteem of the professional public:"—while the *same work* which is *now* so reviled and ridiculed, was *then* executed —"in a most interesting and satisfactory manner." Before proceeding farther, I shall lay before the public a few specimens of the Medical and Physical reviewing.

(No. 1.) 1818.

" In the 4th section, our author enters, first on the consideration of *hepatitis*, of the symptoms of which, he traces all the occasional irregularities with so masterly a hand, that, though his treatment be regulated, it is by no means common, and is entitled to serious attention."—*Med. and Phys. Journ.*

☞ *Compare this with No. 3. a judgment on the same section of my work.*

(No. 2.) 1819.

" In this discussion on the balance of enjoyment and suffering in the different gradations of society, the author, *already so well known and favourably regarded by the profession*, has adduced, in a concise

* The *extra limites* department was originally opened for the purpose of giving authors an opportunity of explaining misapprehensions, or of defending themselves against, what they might conceive to be, unjust criticism in this, as well as other Reviews. As the public must not be charged with these defences, authors are hereby informed, that the expence of insertion is *nine shillings per page*, the exact prime cost of paper and printing. All the control exerted, is over language that might be deemed derogatory to science, or beneath the dignity of the profession.

form, *many interesting and important observations* and reflections, &c. &c.

"One of the most marked characteristics of the whole of Dr. Johnson's works, is derived from a view which he has taken of the system, that, if Darwin be excepted, had, until lately, been but little attended to by English pathologists, &c.

"Now, although this doctrine is not original with Dr. Johnson, yet he has the merit of having displayed it *with accuracy* and precision, *and of having discovered many of its laws.* This is shewn in his work on Tropical Climates, and on *Atmospheric Influence.* We find, too, much interesting matter respecting it in the work before us. It would not be possible to give an adequate view of it in an abstract, and the work itself is drawn up in so concise a manner, as not to admit of an analysis *occupying much less space than the original.*" *Med. and Phys. Journal, Oct.* 1819.

(No. 3.) 1820.

"On the subject of *hepatic* diseases, the author, as on all other occasions when he does not utter *absurdities*, is a mere compiler, and we cannot compliment him on doing this easy duty with much judgment, as he generally selects for his assertions just those which stand most in need of proofs. He takes every thing for granted, which suits his convenience; and if he wanted any thing else, *he would not hesitate to assume it.*" *Med. and Phys. Journal, October* 1820.

It is well known that the critiques of 1819 and 1820, at least, were penned by the *same individual*, and I cannot but envy that individual the luxury of his feelings, on seeing these amiable specimens of his strict integrity, sail on the four winds to every habitable country of the globe.

There are some minor examples of the Reviewer's consistency and veracity scattered through this critique, which cannot fail to entertain the public. Thus in the *first* page of the review, it appears that my work dropped still-born from the press, and was sold by the hundred weight for waste paper; but by the time the critic had got to the *last* page of his review, the work had got to a *third edition*, and was become very profitable to the booksellers! Really the powers of this critick's pen far exceed those of Merlin's wand and harlequin's lath, in transforming every passing object into all the shapes of an evening cloud, and with all the evanescence of an imaginary creation.

Some people may be curious to learn what crime I can have committed, in this short interval, between the critiques, to forfeit my claim upon the approbation of my brethren, and produce this sudden change in the sentiments of my judges. My crime is of a very deep die, and never, I fear, to be forgiven. In 1818, the author of the work traduced resided in a provincial town, and manifested no symptom of rivalry. *Then* he was a very deserving man, and his work was excellent. But having had the audacity to present himself before the public as the conductor of a quarterly journal, he is suddenly transformed to an ignoramus, and his work, so lauded before, is consequently changed into a tissue of absurdities, from alpha to omega!

Nay, at a still more recent period, as the specimens will shew, and before the rival journal emerged from infancy, or occasioned alarm in the breast of the liberal reviewer, *this same reviewer* did, in his critique on "Civic Life," represent the author as *directly the reverse* of what he *now wishes* to make him appear.

"All this (as Dr. Wilson Philip observed upon a similar occasion, and to the same individual) would be quite amusing, were not so gross a violation of the implied compact between a reviewer and the public, too grave a subject for merriment."—*Dr. Philip's Correspondence.* One thing, however, is amply proved by these transactions—the utility of this same competition in periodical journals, and the necessity that existed for an ANALYTICAL REVIEW, governed by some principle of justice, and manifesting some regard for truth. Nothing can be more clear than that the patronage which such a review has experienced from the profession, is the real and secret cause of this torrent of abuse from those who considered themselves possessed of an hereditary and exclusive monopoly in this branch of medical literature.

The Medical and Physical Reviewer has, I am told, stated his determination to write me down, through the medium of the two vehicles abovementioned; for no one fails to perceive the identity of the writer in both. I would recommend to his perusal the following passage in the September No. of the *Journal Complementaire des Sciences Medicales.* "Il serait à desirer que l'on bannit pour toujours des ecrits relatifs aux sciences ces *expressions injurieuses* qui n'ajoutent rien a valeur des argumens, *et qui jettent toujours de la defaveur sur les hommes qui les emploient.*" P. 249.

But to come to some particulars. One would have supposed, that after Dr. Wilson Philip's detection of the misrepresentations and perversions of the Medical and Physical Journal,* the writers in that work would have been a little cautious, at least for a time, of laying themselves again open to public conviction. But that castigation seems to have had only a very temporary effect. Under the prospect of a two months' exemption from exposure, a new review of my work has been put forth, consisting of an uninterrupted chain of garbled quotations, misrepresentations, and perversions. It will hardly be expected that I should follow this enraged Reviewer through all his effusions of envious and jealous feeling; or seriously reply to his ribaldry and ridicule. I shall not be so unwise as to leave the 'vantage ground which I possess, and descend into the arena of abuse, where, in truth, I should stand very little chance of success, with adversaries so well trained to that mode of warfare. I shall merely lay before the public a few specimens of this mode of reviewing, that cannot but have the effect of rousing the attention of every man of reflection towards a practice which but ill accords with the dignity of science and literature, and, on the contrary, which is admirably calculated to degrade the profession itself in the eyes of the public at large.

---

* See the 8th No. of the Medico-Chirurgical Journal, p. 689, *et seq.*

At page 326 of the Journal, in order, I imagine, to make me guilty of something like an absurdity, in supposing a fluid to be stagnant and *oozing* at the same time, the Reviewer coolly adds the *oozing* part himself, and then marks it in *italics*, to render it more conspicuous. The original words (page 73 of my 3d edition) are—"The stagnant blood occasionally finds its way into the intestines, &c." which the reviewer states thus: "The *stagnant* blood, *oozing* from the liver, finds its way, &c." thus gratuitously throwing in the words, "*oozing* from the liver," in contradistinction to its *stagnancy!* It is true that I have made use of the word "*oozing*;" but then it was in the *dissection* of a gorged liver, where blood *oozed* from all points, "at each cut of the scalpel." Such trifling amalgamations of dead and living matters are nothing, it seems, in the refulgent flashes of this gentleman's witty criticisms.

In page 324, the Reviewer's wit knows no bounds. "The author, says he, has forgot to *exclaim* how *remarkable* it is for an *inclination to stool* to take its seat *in the stomach*, and then fly to the *chest.*" It would be much more natural for me to exclaim how *remarkable* it is that a Reviewer, who pretends to impartiality, cannot read a single passage without misrepresenting its meaning. I never stated that the *inclination to stool* flew to the stomach and chest, but that the *irritation* which produced it, did so, as any man of common apprehension and impartiality would construe the sentence. "The first warning symptom (in spasmodic asthma) is often a sudden inclination to stool, or intestinal *irritation which* quickly shifts its seat to the stomach, and then flies to its favourite station, the chest."

In the same page of the Journal, my total ignorance of all pathological matters is demonstrated in the following manner. I am there stated to assert, that "disorders of the internal viscera, as the liver, *stomach*, &c. are not inflammatory, *unless produced by climate.*" If the reader will take the trouble to turn to the passage in my work, he will probably be surprized, (if any thing can surprize him on this occasion) to find that I have stated just the *reverse* of the above; namely, "that those disordered states of the stomach, *resulting from atmospherical impressions on the surface*, do *not* often partake of an inflammatory nature." *P. 28. 3d Edit.*

What will the profession think of a public journalist who can thus deliberately reverse the sentiments of an author, in order to hold up these his misrepresentations to ridicule? No author's character is safe, while such reviews are countenanced. And if a reviewer will publicly send forth such calumnies against a man who has the means of circulating their antidote to a far greater extent than the poison can reach, what protection has the defenceless victim of slander and misrepresentation, in the hands of such unprincipled critics? It is high time for the public to look to this.

The whole critique, in fact, is a tissue of this kind of misconstruction or misrepresentation.* But the Reviewer has suffered himself

---

* I appeal to a writer, whom the Reviewer will not accuse of partiality, for the truth of this assertion. The Medical Intelligencer of last month passes the following sentence on the critique in question:

to be carried forward with such impetuosity by these passions, that he exposes his lack of knowledge or principle at every step. Thus the universally well known experiments and observations of Bichat and Saunders on the biliary secretion and digestion, are triumphantly held up to derision as *my fancies*, for which it would be as vain to look for *evidence*, "as to expect blood from a flint." Yet these *fancies* are the direct results of ocular demonstration and of reflection, recorded by the abovementioned authors—authors whose works the medical and physical Reviewer surely must be acquainted with. Certain citations, too, which I have made from Laennec, *an author recently reviewed by the critic himself*, are held up to public ridicule as *my* puerilities. In short, the above and other late criticisms have exemplified all that ever was set down, even in caricature, of the licence of some critics. No one will deny, after this, how necessary is a reform in this department of medical literature. The eyes of the public, indeed, must now be open to the shameful delinquencies of the journal in question, whose conductors are certainly taking the most effectual means of advancing the interests of those journals of which they are so thoroughly jealous.

It is thus that malice often overacts its part, and injustice works its own downfal. Henceforth let no man plume himself with the praises of the Medical and Physical Journal, or clothe himself in sackcloth and ashes at being condemned by a tribunal remarkable only for its utter disregard of truth and total want of judgment.

I shall next proceed to prove this reviewer guilty of the most illiberal misrepresentation that can well be imagined. He states at page 325, that the instruction given by me in dysentery, two years ago, in the second edition of my work, was—"the exhibition of submuriate of quicksilver, in scruple doses, twice or three times a day, without any other medicines." He insulates this passage and carefully suppresses both what precedes and follows it. *Preceding* it I have distinctly stated that—"the practice I would recommend, from much experience, is, the introduction of mercury, in comparatively small doses, either alone or combined with an opiate, or, which is preferable, with an opiate and diaphoretic," &c. p. 46. The passage *itself* I have stated thus:—"I am now to mention a practice which, *in some cases of emergency*, I tried with unexpected success (in the East Indies) and which was tried by a few others, without any communication of ideas, and with similar results. It consisted in the exhibition of submuriate," &c. *2d ed. p. 47. Succeeding* this passage the reader will find it thus:—"I may add that I have, *in several instances*, pur-

---

"The pages of literary history have been too often stained by the passions or prejudices of opposing minds; but such "*damned spots*" ought not to be multiplied at a period when the progressive increase of knowledge requires a correspondent liberality of sentiment. Whatever provocation the learned and able editor of this (the Medical and Physical) Journal may have felt, this we wish, that he had not inserted (he might have said *written*) this review, which, in exaggerating the faults, and omitting the excellencies, of Dr. Johnson's work, does not, we conceive, fulfil the purposes of legitimate criticism."

sued the same plan in dysenteric cases in this country, with much felicity of result. I did *not* indeed adopt this practice generally, being quite satisfied, *in ordinary circumstances*, with the plan before detailed. But whenever I had occasion to push boldly on for ptyalism, *in cases where time appeared extremely precious, and when the symptoms were such as indicated the danger of rapid destruction of organization internally, I hesitated not to exhibit calomel in scruple doses, &c.*" P. 48.

I believe the annals of literature could not afford a more flagrant breach of justice, or a more garbled misrepresentation, than the reviewer has here set forth to the public. But the profession are not destitute of discernment, and they will easily appreciate the tenor and object of such reviewing.

It is only necessary further to state, that this section on dysentery, in the second edition of my work, being a literal transcript, or nearly so, of that in my work on tropical climates, I carefully revised it in the third edition, and adapted it, as stated in the preface, as nearly as I could to the climate in which we live. In a new edition of my work on Tropical Climates, now in the press, the original passages stand as they were first written, for I have no occasion to alter facts, or blot out a single line of what I set down from observation or conviction.

And here I may observe, that this experienced reviewer has not thought proper to attack a single doctrine or practice in my whole work, by fair argument; and that the only weapons he uses are those of ridicule, founded principally on falsified quotations.

In parting with my liberal, impartial, and veracious Reviewer, I cannot help condoling with him on the tone of pathetic lamentation with which he concludes his elegant diatribe. It appears that the intellect of the profession is labouring under a temporary hallucination; that *some* writers have got the ear of the public who are absolutely below mediocrity; while the productions of *others*—men of sublime talents—"superior workmen," are suffered to pine in obscurity, though a future age will do them justice, and must be astonished at *their* genius, and the want of discrimination in the public! This is really a melancholy sign of the times. Only think how provoking it is, that the profession should perversely remain insensible to the transcendant merits of the Medical and Physical Journal, and to the Bœotian stupidity of the Medico-Chirurgical Review.

But I would recommend my friend the Reviewer to have courage and perseverance. Repeated effusions of such edifying criticisms as he has lately produced,* must ultimately attract universal attention to the unfathomable sources of knowledge, probity, candour, and impartiality, whence they flow. In charity towards my incapacity the Reviewer has *disinterestedly* advised me to write no more. In profound admiration of his splendid talents, and distinguished liberality, I earnestly exhort him to write on. He cannot confer a greater obligation on his humble servant, JAMES JOHNSON.

---

* See Dr. Halloran's letter, in the 8th article of this number; Dr. Philip's detections, &c. &c.

